# A rational route to transformative decisions

**DOI:** 10.1007/s11229-021-03432-w

**Published:** 2021-10-20

**Authors:** Daniel Villiger

**Affiliations:** Institute of Philosophy, Zollikerstrasse 117, 8008 Zurich, Switzerland

**Keywords:** Transformative experiences, Decision theory, Higher-order facts, The shark problem, Negativity bias

## Abstract

According to Paul (Transformative experience, 1st edn, Oxford University Press, 2014), transformative experiences pose a challenge to decision theory since their value cannot be anticipated. Building on Pettigrew’s (in: Lambert, Schwenkler (eds) Becoming someone new: essays on transformative experience, choice, and change, Oxford University Press, pp 100–121, 2020) redescription, this paper presents a new approach to how and when transformative decisions can nevertheless be made rationally. Thanks to fundamental higher-order facts that apply to any kind of experience, an agent always at least knows the general shape of the utility space. This in combination with the knowledge about the non-transformative alternative in the choice set can enable rational decision-making despite the presence of a transformative experience. For example, this paper’s approach provides novel arguments for why gender transition (cf. McKinnon in Res Philosophica 92(2):419–440, 2015) or staying childfree (cf. Barnes in Philos Phenomenol Res 91(3):775–786, 2015) can be rational.

## Introduction

In her influential book *Transformative Experience*, Laurie Paul (2014) raises the question of whether you can rationally choose an option that you have never chosen before, such as becoming a parent. Her argument is as follows: since you have not yet experienced what it means to become a parent, you cannot assess the option’s utility. Such understanding is only possible when you undergo this experience, which is why the choice is transformative. Here, the transformative character of such a decision can be twofold. On the one hand, your knowledge can be expanded by undergoing a new experience. This would be an epistemic transformative experience (which will be the main focus of this paper). On the other hand, undergoing a new experience might change your preferences. This would be a personally transformative experience. Of course, these two types of transformative experience can also occur in combination.

Several authors have already suggested ideas about how and when transformative decisions could still be made rationally (e.g. Campbell, 2015; Dougherty et al., 2015; Kauppinen, 2015; Pettigrew, 2015; Reuter & Messerli, 2018; Sharadin, 2015). Among these, Pettigrew’s (2015, 2016, 2020) approach is particularly promising because it is the only one that is closely tied to expected utility theory and applicable to all (epistemic) transformative decisions.^1^ Moreover, his solution includes a suitable decision-theoretical formalisation of transformative outcomes. So, Pettigrew definitely goes in the right direction, yet, as it turns out, his solution involves a hardly solvable technical difficulty which makes it more or less inapplicable.

To address this difficulty, this paper presents a novel approach to transformative decisions that builds on Pettigrew’s formalisation.^2^ In short, although a decision-maker is not able to determine the precise value of a transformative outcome, she can use higher-order facts to assess its range of relevant utility values. Sometimes, there are closely related higher-order facts that enable the determination of one endpoint of this range (e.g. getting your legs amputated without anaesthesia) or even both endpoints (e.g. eating durian). However, in other decision situations, there are no higher-order facts that make it possible to set an endpoint (e.g. becoming a parent). This is where two fundamental higher-order facts about experiences come into play by informing the decision-maker about the general shape of the utility space. Due to this information, rational decision-making can become possible, depending on the other alternatives that are part of the choice set. Accordingly, this paper’s approach demonstrates the importance of an often-neglected aspect of transformative decisions: the consequence of the non-transformative option, which normally goes along with doing nothing and thus sticking to the status quo.

The paper is structured as follows: Sect. 2 quickly recaps Paul’s utility ignorance objection. Section 3 presents Pettigrew’s solution to the problem of transformative decisions and its insufficiencies. Section 4 discusses whether and how the range of relevant utility values of transformative outcomes can be assessed. Building on that, Sect. 5 then shows how specific combinations of transformative and non-transformative alternatives in a choice set enable rational decision-making.

## Paul’s utility ignorance objection

First of all, the challenge of transformative experiences mainly arises within a specific understanding of decision theory, namely deliberative non-constructivist realism.^3^ This means that (1) utility and credence are real mental states (Buchak, 2013); (2) facts about an agent’s utilities can extend beyond her preferences;^4^ and (3) not only the outcome of the chosen option is important but also the deliberation that led to choosing it (Pettigrew, 2020).

Next, let us briefly review how transformative experiences challenge such an understanding of decision theory. In order to decide which of multiple options to choose, we compare the expected utility of these options and then choose the option (or one of the options) with the highest expected utility. We assess the expected utility of the options that are part of our choice set as follows: (1) we select one of the multiple options; (2) we determine the utility of each of its potential outcomes via our utility function; (3) we multiply each of these utilities with the probability of the respective potential outcome via our credence function; (4) we add up all these values to obtain expected utility; and (5) we repeat this process for all the options in our choice set. It is the second step that is problematic in the case of transformative experiences. According to Paul (2014, p. 26f), when we consider our options, we evaluate each possible outcome that they involve by imagining or running a mental simulation of what it would be like if that outcome were to occur. However, this simulation is impeded in the case of transformative experiences because we cannot know what it would be like to experience a certain outcome if we have never experienced it before. In other words, we do not know the phenomenal character or subjective value of the experience. As a consequence, we cannot determine the utility of the transformative outcome and, therefore, cannot evaluate the expected utility of the option.

Importantly, the calculation of a transformative outcome’s utility is only partly impeded, namely in the case of its subjective value. The subjective value comprises the ‘ways we’d experience ourselves in such outcomes’ (Paul, 2015a, p. 514). In contrast, the non-subjective value of a transformative outcome, which includes its non-experiential aspects, can be assessed. However, this is only of some help, as Paul (2015c, p. 165) notes that even if other features of an outcome are relevant, the value of the phenomenal outcome might be so positive or so negative when it occurs that none of these really matter. So, we can never know whether the unknown subjective value of an outcome will swamp its non-subjective value (Paul mentions some exceptions, as we will see later). Still, since non-subjective values can be assessed in general, we will focus on options’ subjective values in this paper’s analysis. In so doing, we assume that options’ non-subjective values do not swamp their subjective values and therefore can be left out in the decision-making process.^5^ Thus, from now on: (1) when I speak of the utility of an outcome, I refer to the utility that goes along with the outcome’s subjective value; and (2) the option with highest subjective value is also the option to be rationally chosen.

## Pettigrew’s fine-graining response and its problem

Pettigrew (2015, 2016, 2020) offers a straightforward solution to the problem of epistemic transformative experiences. He expands the uncertainty about which one of the possible outcomes occurs if you choose option X by how much utility you will derive from this potential outcome. Let us exemplify this by means of the decision whether or not to become a parent. We assume that staying childfree is not a transformative experience, and thus we can easily calculate the expected utility of that option. Moreover, for simplicity, we assume that if we choose to become a parent, we become a parent. Therefore, the option {trying to become a parent} only has one general outcome: being a parent. Since this outcome involves a transformative experience, we cannot know its subjective value or the utility it comprises. This is where Pettigrew’s solution comes into play. We subdivide the general outcome of being a parent into many more fine-grained outcomes that directly comprise the utility of being a parent. For example, one possible outcome could be {being a parent and utility of being a parent is 12} or {being a parent and utility of being a parent is − 5}. Self-evidently, the utility of the outcome {being a parent and utility of being a parent is 12} is 12 and that of {being a parent and utility of being a parent is − 5} is − 5. Next, we determine how probable each of these fine-grained outcomes is via our credence function and thus attain the expected utility of the option {trying to become a parent}. In this way, we can compare the expected utility of {staying childfree} with that of {trying to become a parent} and then choose the option with greater expected utility. Pettigrew (2020) calls this the ‘Fine-Graining Response’ and states that it seems to answer Paul’s ‘Utility Ignorance Objection’.

Even though Paul (2015b) argues in her first reply to Pettigrew (2015) that this redescription eliminates an important aspect of transformative experiences, namely the phenomenal characters of such experiences, Pettigrew (2020, p. 106) later writes that Paul allows the redescription. Nonetheless, there is one crucial aspect on which Paul vehemently disagrees with Pettigrew: on what sort of evidence we might base our credences of fine-grained outcomes.

Of course, the question of how to set the credences of fine-grained outcomes is of utmost importance in Pettigrew’s framework since these credences completely determine an option’s expected utility.^6^ In fact, we could say that an option involves all existing utility values and that our credences determine which are relevant (i.e. having positive probability) and how relevant (in the sense of probable) they are.^7^ Pettigrew (2020) proposes to use the testimony of people who have already undergone a given transformative experience in order to determine the credences of utilities. For instance, if 70 out of 100 people who became parents say that being a parent involves a utility of 12, 20 say that it involves a utility of 2, and 10 say that it involves a utility of − 5, we could obtain the following credences:

- P of {being a parent and utility of being a parent is 12} = 0.7
- P of {being a parent and utility of being a parent is 2} = 0.2
- P of {being a parent and utility of being a parent is − 5} = 0.1

This would lead to an expected utility of 12 * 0.7 + 2 * 0.2 + (− 5) * 0.1 = 8.3, which we can then compare with that of another option. Importantly, Pettigrew does not claim that decision-makers ought to determine their credences in such a strict way but simply that they can assess their credences by using others’ testimony.^8^ Still, he does not offer another method for assessing utility values and credences.

Paul (2015b) criticises this method of determining credences. According to her, an agent who reaches a transformative decision in such a way would be alienated from that decision. It leads to an inauthentic choice because we do not sufficiently consider our own utilities, but rather those of others, thereby eliminating the first-person perspective from our choice. Ultimately, such a procedure could force people to engage in a form of self-denial in order to be rational decision-makers. In turn, this would promote a future determined by big data or so-called ‘big morality’ rather than by personal deliberation and authentic choice.^9^

Admittedly, this sounds rather dystopic and seems to condemn Pettigrew’s solution. However, on the one hand, it has to be mentioned that authenticity is not per se a criterion of rational choice and is thus insufficient to prevent rational decision-making. On the other hand, Pettigrew (2015) counters that the procedure of setting credences via testimony does not completely ignore the decision-maker’s own utilities. On the contrary, the statistical evidence concerning how much utility others derived from an option precisely tells a decision-maker something about her own utilities. This argument appears reasonable. Let us analyse the consequence if an agent were not allowed to use statistical evidence. She would have no idea which utility values were more probable than others and thus would have to apply the Principle of Indifference, which implies that every utility value is equally likely. Pettigrew (2016) also mentions this idea:

If I have no information about the experience of becoming a parent, I might perhaps follow the Principle of Indifference and divide my credences equally over it being wonderfully fulfilling, quite enjoyable, quite unenjoyable, or awful (on the supposition that I become a parent). (p. 931)

However, while Pettigrew only lists four fine-grained outcomes, there could be many more (theoretically up to infinity). Since this procedure does not give up the first-person perspective, it should be authentic. However, it is obvious that such an authentic agent knows much less about her utilities than one who uses statistical evidence. This is because a uniform probability distribution on all existing utility values is less likely to anticipate her actual utility value than statistical evidence that is well-matched with her personality.

It may seem that Pettigrew’s fine-grained response in combination with testimony solves the problem of epistemic transformative experiences. Unfortunately, this is not the case. There is a major problem that involves the derivability of utility values from testimony. Since the challenge of transformative experiences suggests a deliberative non-constructivist realist understanding of decision theory, utilities should be defined on interval scales. Otherwise, expected utility theory would not be applicable as a tool for deliberation. In turn, this implies that a positive affine transformation does not affect the properties that are relevant in a utility function (Isaacs, 2020). For example, it does not matter if we say that options A, B, and C yield utilities of 1, 2, and 3 or if we say that they yield utilities of 15, 25, and 35.

This is problematic because it makes testimony about utility values useless unless there are anchors to which everyone can refer. As Isaacs (2020) argues, a comparison between two utility functions is only meaningful if there are two distinct anchors between them. These anchors enable the determination of the relative positions and the relative scales of the two utility functions and thereby capture their full structure. However, from a practical perspective, Isaacs is sceptical whether this approach is feasible because it is doubtful whether such anchor utilities (always) exist. At least, they are most certainly absent in usual statistical evidence. This constitutes an important limitation of Pettigrew’s fine-grained response. A possible way out would be to abandon testimony and reintroduce the Principle of Indifference. Then again, a coarse-grained outcome could theoretically lead to any existing utility value. We are left with a difficult question: how do we assess the range of relevant utility values?

## Assessing the range of relevant utility values

Although a transformative experience makes it essentially impossible to calculate the subjective value of an option that inheres it, there are exceptions to this rule. For example, Paul (2014, p. 27) notes that an agent can know that being eaten by a shark will be horrible, even if she does not determine what it would exactly be like via running cognitive simulations. Thus, for the situation of being eaten by a shark, we know that every possible outcome is bad, regardless of how fine-grained these outcomes are. Campbell and Mosquera (2020) infer from this that there might also exist the opposite of such ‘sharky outcomes,’ which they call ‘heavenly outcomes.’ Heavenly outcomes would imply that every possible outcome is good, regardless of how fine-grained these outcomes are. Dougherty et al. (2015) make a similar point when they speak of real-world examples of uniformly positive testimony. For instance, for professional tennis players, winning their first grand-slam title might be such a heavenly outcome.

In order to describe heavenly and sharky outcomes within Pettigrew’s (2020) framework, we set the following anchor: the utility value of zero goes along with experiences that, overall, are neither positive nor negative and therefore can be described as being neutral.^10^ As a consequence, any good experience has greater than zero utility, and any bad experience has less than zero utility. In the case of sharky outcomes, we know the upper endpoint of the range of relevant utilities, which is below zero. This tells us that all relevant utilities are negative even if we might not know the lower endpoint of the range. In other words, we can say that sharky outcomes definitely involve negative valence.^11^ Conversely, in the case of heavenly outcomes, we know the lower endpoint of the range of relevant utilities, which is above zero. Therefore, all relevant utilities are positive even if we might not know the upper endpoint of the range, and we can say that heavenly outcomes definitely involve positive valence. This knowledge about an outcome’s valence can make rational decision-making possible, as Paul (2014) proposes for the case of ‘decisions like the decision to avoid swimming with sharks’ (p. 28).

The apparent existence of sharky and heavenly outcomes opens a door for restricting the range of relevant utility values. Yet, the consequence that there are both non-assessable transformative outcomes and sharky and heavenly transformative outcomes appears to be problematic. Campbell and Mosquera (2020) argue that their conjunction leads to an implausible discontinuity in the evaluability of outcomes, which they call ‘the shark problem.’ Might the shark problem close our just-opened door for restricting the range of relevant utility values?

I do not believe it does. Let us examine the authors’ argument more closely. The example that they use to pose the shark problem involves different durations (in seconds) for which an agent will have to endure intense pain within 30 days. They then set an (arbitrary) border that defines normal and sharky outcomes: Everything up to 500,000 s of intense pain within 30 days involves a normal outcome, meaning that we cannot know the subjective value of that outcome before experiencing it. In contrast, everything above 500,000 s of intense pain within 30 days involves a sharky outcome. In this example, the border between normal and sharky outcomes truly appears to be problematic. Why should we be able to compare 500,000 with 500,001 s of intense pain but not 1 with 500,000 s of intense pain? It appears that Campbell and Mosquera have a point. Yet, the actual problem of their example is not so much the existence of such a border in and of itself, but where they place it. More precisely, the border that determines normal and sharky outcomes in regard to intense pain should lie between 0 and 1 s. Experiencing intense pain is intuitively bad, no matter how long it lasts. Contrary to that, experiencing no pain is a neutral outcome.^12^ As a consequence, there is a natural border between experiencing intense pain for 0 and 1 s.

Let us illustrate this natural border with the following example of a decision. Should I stick a scriber into my thigh or not? As a matter of fact, I have never stuck a scriber into my thigh, and so I do not know the subjective value of this experience. Thus, might every existing utility value be relevant in regard to this outcome? I would clearly say no. Only utility values that are below zero are relevant here. As a consequence, I know that the experiential valence of sticking a scriber into my thigh is negative. Moreover, I know what it is like not to stick a scriber into my thigh and would describe it as having neutral valence, meaning it yields zero utility. Due to the different valences of my two options, I can rationally prefer not sticking a scriber into my thigh to sticking a scriber into my thigh.

Of course, the same is not possible in the case of an outcome with ambiguous valence. Ambiguous valence means that the lower endpoint of the range of relevant utility values is below zero, whereas the upper endpoint is above zero. Referring to the terminology of Campbell and Mosquera (2020), we call an outcome that fulfils these requirements a normal outcome. For example, being a parent could either involve a good or a bad experiential outcome, meaning a utility value smaller or larger than zero. Therefore, we are not able to say whether the expected utility of being a parent is smaller or larger in comparison to not being a parent (which we assume to be zero for the time being).^13^ When Paul (2014) talks about the problems that transformative experiences pose to decision theory, she solely refers to such normal outcomes with ambiguous valence. In so doing, she assumes that we can evaluate whether the valence of a transformative outcome is ambiguous or unambiguous. The fact that we can do that does not contradict the general circumstance that we cannot assess the precise subjective value of an outcome that we have never experienced before. To put it differently, uncertainty about fine-grained utility values is compatible with certainty about the coarse-grained range of relevant utility values. In turn, our ability to assess whether the valence of a transformative outcome is ambiguous or unambiguous seems to solve what Campbell and Mosquera (2020) call the shark problem: sharky or heavenly outcomes involve unambiguous valence, whereas normal outcomes involve ambiguous valence.

Sharky and heavenly outcomes demonstrate that, at least sometimes, people can set at least one endpoint of an outcome’s range of relevant utility values. What gives them this ability? The answer to this question can be found by considering normal outcomes. Since we defined that a neutral experience has a utility value of zero, normal outcomes could theoretically lead to any existing utility value between negative and positive infinity. Yet, Paul (2014) says that sometimes we can restrict this range, and uses the durian fruit whose taste is very unique as an example. She writes:

We can know that certain types of new experiences won’t affect us that much, and so, for that sort of new experience, we can know the approximate range of possible subjective values. The way we grasp the range of the values isn’t by knowing what it would be like to have the experience, but by knowing enough about a higher-order fact about that type of experience to know that there is a low limit on how positive a value for that experience could be (and a low limit on how negative the value could be). Trying durian seems to be like this—after all, it’s just trying a new kind of fruit. You’ve had fruit before. How good (or bad) could the experience of tasting this new fruit be? (p. 37f)

We see that it is the knowledge of a higher-order fact about the type of transformative experience that enables us to assess its range of relevant utility values. Moreover, while Paul illustrates this by means of a normal outcome (eating durian), it is not far-fetched to assume that the same procedure also leads to the identification of sharky and heavenly outcomes.^14^

The idea behind higher-order facts stems from work in cognitive science on how humans are able to make accurate and flexible predictions about unknown situations (e.g. Tenenbaum et al., 2006, 2011). In short, we use inductive inferences which draw on abstract knowledge about higher-order similarities between the novel situation and already experienced situations. Therefore, by categorising experiences, we abstract higher-order structures from them. For example, while eating an apricot and eating a nectarine do not lead to the same first-order experience, there are still many higher-order similarities between them: both are sweet and slightly juicy, have a similar firmness to the bite, and can be eaten directly. Because of that, the first-order categories ‘eating apricot’ and ‘eating nectarine’ are part of a higher-order category, such as ‘eating stone fruit’.^15^ The facts about this higher-order category can then be used to map out higher-order properties of novel experiences, as for example eating a plum. At this, higher-order facts will not reveal the exact first-order experience of eating a plum but rather its general character. Still, this knowledge can be sufficient to approximately determine the subjective value of eating a plum. In so doing, we cognitively model an outcome where the plum has the same higher-order properties as the stone fruits that we have eaten in the past, in virtue of its membership in the stone fruit category. Such a process can be represented using computational modelling, where higher-order categorisation (eating stone fruit) guides our reasoning about a novel lower-order case (eating plum).^16^

The method to assess the subjective value of a novel experience based on the higher-order properties that it shares with our previous experiences is persuasive. Yet, it leaves an open question: how do we know which (if any) higher-order features a novel experience shares with our previous experiences? To return to the durian example, when seeing an unopened durian, we can only infer that it is a fruit which looks a bit like a greenish lychee in the size of a pineapple. But which possible properties of fruits does durian have? Maybe it is a poisonous fruit, leading to an extremely low utility value when eating it. Or maybe it is a hallucinogenic fruit, causing a psychedelic trip when eating it, with the potential for an extremely high utility value. So, just knowing that durian is a fruit does not really restrict the range of relevant utility values.^17^ For that, we need some sort of testimony (or description) about eating durian that indicates which higher-order properties of eating durian are relevant in determining its subjective value. If we then know these relevant higher-order properties from previous experiences, we can base our cognitive model of eating durian on them and assess its subjective value.^18^ Therefore, if testimony indicates that durian is edible, has a foul smell, and a taste that some find delicious and others disgusting, we can think of previous experiences that involved these possible higher-order features of eating durian. In turn, these previous experiences help us to restrict the range of relevant utility values of eating durian. To put it differently, we have had delicious and disgusting food before; how high or low can the subjective value of eating durian possibly be?

So, higher-order facts enable us to roughly assess the subjective value of eating durian despite it being a transformative outcome. But what about experiences that are more far-reaching such as becoming or being a parent? In that case, it is difficult to find a higher-order fact that sufficiently represents becoming a parent in order to restrict the range of relevant utility values. Possible higher-order facts could be how much you like children in general or, given that some of your friends or relatives have small children, whether you like to spend time with them. Then again, these are not your children, which could make a huge difference. Another higher-order fact might be how much you enjoy spending time with your own family. Yet, you will have a completely different role when you start your own family, and there is no guarantee that the family climate within your ‘original family’ and your ‘founded family’ will be similar (which can be good or bad), relativising this higher-order fact. For pet owners, a different higher-order fact might apply, namely whether they like to take care of and be responsible for another being. However, taking care of a dog is, of course, not the same as taking care of your child. Therefore, this higher-order fact is also not really appropriate.

In fact, it seems that no higher-order fact is able to help us define the range of relevant utility values regarding the outcome of becoming a parent. Paul (2014) agrees that such situations can occur, and poses a more general challenge that the usage of higher-order facts faces:

The problem derives from the fact that, while we can use higher-order cognitive modeling to assess the higher-order subjective value of novel experiences, because the experience is epistemically transformative, we cannot know which, if any, of the experiences of higher-order features that have contributed to the subjective value of previous experiences will carry over to the values we assign to outcomes of the epistemically transformative experience. (p. 164)

But might there be higher-order facts that are applicable to any novel experience, meaning higher-order facts that are completely independent of the specific type of experience? I think there are two: finite range of relevant utility values and negativity bias. Let us discuss these two fundamental higher-order facts about experiences one by one.

The first fundamental higher-order fact comprises that the range of relevant utility values is bounded by possible experience and therefore finite. This can be derived as follows: A utility value of either positive or negative infinity implies that experiences can endlessly get worse or better. Thus, no matter how good are bad an experience is, it can always get better or worse. This is not tenable because an agent’s life is finite. In other words, even if you are in a negative spiral where experiences get worse and worse, this will not go on endlessly, because sooner or later you are dead. As a consequence, regarding the range of relevant utilities, the lower endpoint cannot be negative infinity and the upper endpoint cannot be positive infinity.

The second fundamental higher-order fact refers to whether the lower and upper endpoints of the range of relevant utility values are equally distant from zero. As defined above, an agent’s point of zero utility goes along with experiences that, overall, the agent evaluates as being neither positive nor negative and therefore as being neutral. The question now arises whether, starting from this point of zero utility, potential experiences can be as positive as they can be negative (and vice versa). To put it differently, do humans have the same capacities for the positive and the negative?

This seems not to be the case. Already Schopenhauer (1966) noticed the imbalance between the potential of positive and negative experiences when he wrote: ‘We feel pain, but not painless. … We feel the desire as we feel hunger and thirst; but as soon as it has been satisfied, it is like the mouthful of food which has been taken, and which ceases to exist for our feelings the moment it is swallowed.’ (p. 575). So, the human body is geared to negative sensations. For instance, while nociceptors innervate skin, joints, muscles, and internal organs (Julius & Basbaum, 2001), there is no such widespread parallel system detecting pleasurable stimuli. The reason behind this is not far to seek: our survival arguably depends much more on urgent attention to potential dangers than on seizing opportunities for positive experiences (Baumeister et al., 2001). In line with that, it is much easier to experience intense long-lasting pain and suffering (e.g. via an accident, disease/illness, or torture) than intense long-lasting pleasure and happiness. This circumstance gets also supported by the fact that people hedonically adapt to positive experiences faster and more completely than to negative experiences (Lyubomirsky, 2011). Finally, we can effortlessly think of sharky outcomes such as being raped or being hit by a car but it is much more difficult to think of outcomes that are universally heavenly. For instance, even winning the lottery—an outcome that many dream of—has ruined some people’s life (Chan, 2016).

In the psychological literature, the circumstance that humans have larger capacity for the negative than for the positive is represented by the so-called negativity bias. Jordan (1965) provided the first evidence for it when he examined the symmetry of liking and disliking in psychological scales. Such scales often include a neutral midpoint and two equidistant endpoints, as for example ‘like very much’ and ‘dislike very much.’ Here, Jordan found that the subjective distance between the midpoint and the upper endpoint was shorter than between the midpoint and the lower endpoint. Since this first study conducted by Jordan, much more research has revealed the human tendency for a negativity bias (for reviews, see Baumeister et al., 2001; Rozin & Royzman, 2001). This has been observed in the case of attention (Öhman et al., 2001; Pratto & John, 1991), information weighting (Gilovich, 1983; Peeters & Czapinski, 1990), decision-making (Kahneman & Tversky, 1979, 1984; Ruggeri et al., 2020; Tversky & Kahneman, 1991), and emotions (Baumeister et al., 1994; Esses & Zanna, 1995).^19^

Altogether, these findings imply that from a fundamental higher-order perspective, we can generally say that the lower endpoint of relevant utility values is further from zero than the upper endpoint. While this information does not reveal the intervals between the two endpoints and the zero point (which differ between agents anyway), it indicates that the general shape of the utility space is tilted towards the negative.

Each of these two fundamental higher-order facts can be sufficient to enable rational transformative decision-making. Whether they are sufficient exclusively depends on the agent’s alternative option(s); an often-neglected component of transformative decisions. Therefore, let us more closely examine the role of a non-transformative option in transformative decision-making and how it interacts with the two fundamental higher-order facts.^20^

## The importance of the alternative

In order to illustrate the role of a non-transformative option alongside a transformative option, we return to the decision of whether or not to eat durian. Let us imagine an agent named Ann who sometimes suffers terrible migraine attacks that last several days. One trigger is a low level of blood sugar. Yet, Ann can prevent a migraine attack from low blood sugar if she quickly eats something after she notices that her blood sugar has dropped. Let us imagine that Ann is strolling around in the backcountry of Indonesia and suddenly realises that her blood sugar is low. Unfortunately, she has no food with her and there are also no supermarkets or restaurants nearby. However, she spots a fruit stall that only sells durian. She quickly goes there and the merchant offers her a durian, about which Ann has already read in her travel guide. What are the specifics of this decision situation? On the one hand, Ann is able to roughly assess the range of relevant utility values that eating durian involves due to the description in her travel guide and higher-order facts (following Paul, Ann has had fruit before—how good or bad could the experience of tasting this new fruit be?). This assessment reveals that eating durian could lead to a positive or negative outcome. On the other hand, Ann knows the outcome of not eating durian which is negative, since it comprises a terrible migraine attack lasting several days.

We consider two possible configurations. The first is that every possible outcome of eating durian is better than the outcome of not eating durian. This implies that the lowest relevant utility value of eating durian is still above the utility value of not eating durian (and as a consequence thereof suffering a migraine attack). Thus, Ann can rationally choose to eat durian. The second configuration entails that some possible outcomes of eating durian are worse than the outcome of not eating durian. More precisely, eating durian can be so grouse that it causes Ann to vomit which makes her blood sugar situation worse. She knows from a previous instance that this would lead to a more severe migraine attack. However, it is only slightly more severe. Because of that, the lowest relevant utility value of eating durian is still closer to the utility value of not eating durian than the highest relevant utility value of eating durian. Ann then applies the Principle of Indifference and therefore assigns all utility values the same probability.^21^ As a result, the expected utility of eating durian turns out to be higher than that of not eating durian which is why Ann can rationally choose to eat durian.

Let us contrast this first scenario with a second one. Ann is strolling around in an Indonesian town and passes a fruit stall with a merchant who offers her a durian—a fruit about which she has already read in her travel guide. Since she is neither hungry and nor curious about how durian tastes, we assume that not eating durian involves a neutral outcome and therefore a utility value of zero. In contrast, eating durian comprises an outcome with ambiguous valence which Ann can roughly assess by means of the description in her travel guide and higher-order facts. At best, eating durian leads to a delicious fruit experience, whereas at worst, it causes her to vomit. Because Ann knows that vomiting is relatively more negative than having a delicious food experience is positive, the lowest relevant utility value of eating durian is further from zero than the highest relevant utility value. Applying the Principle of Indifference, she then assigns all utility values the same probability. Consequently, the expected utility of eating durian turns out to be lower than zero which is why Ann can rationally choose not to eat durian.

The two different scenarios show that the consequences of the non-transformative option determine whether the transformative option is chosen or not. This leads to an important insight: when studying transformative decisions, we should not only examine the transformative option but also its alternative. With this in mind, let us in a next step depict when an agent is able to rationally reach her decision even though she is restricted to fundamental higher-order facts. There are two possible starting situations for rational transformative decision-making in such cases: (1) the expected utility of the non-transformative option is close to or even at the threshold below which life is no longer worth living; and (2) the expected utility of the non-transformative option is equal to or greater than zero. The following paragraphs illustrate this in more detail by means of two real-world examples: gender transition (for case 1) and becoming a parent (for case 2).

McKinnon (2015) analyses gender transition from a transformative experiential perspective. Gender transition most certainly involves both an epistemic and a personal transformation. However, for the moment, we consider mainly the epistemic transformation and later quickly consider the personal transformation as well. In order to formalise the decision whether to transition or not, McKinnon reduces the outcome space to four outcomes: {transition and happy}, {transition and unhappy}, {not transition and happy}, and {not transition and unhappy}. She then writes that in the case of many trans people, the outcome {not transition and happy} is so unlikely as to be effectively impossible. This is because they know from experience that non-transition goes along with deep unhappiness; often, non-transition means depression, suicidal thoughts, and even suicide attempts. Therefore, many trans people actually must decide between non-transition and living an unhappy life or transition and living a happy or unhappy life, with unknown probabilities for the latter two outcomes. Moreover, as mentioned above, non-transition is even more dire for some trans people since it drives them to suicide. According to McKinnon (2015, p. 424), those who take their life have frequently reported that when they came to the realisation (or strong belief) that they could not transition, often because of a lack of family and social support and acceptance, they perceived no other future than one of living in misery as their birth-assigned gender. Therefore, for these people, it is either non-transition and suicide or transition and the possibility of a better life.

McKinnon says that in situations where non-transition leads to a miserable life or even suicide, choosing transition has to be rational because, while it hardly makes the situation worse, it has the chance to make it better.^22^ However, she also states that she makes no comment on how decision theory should account for cases structured such as gender transition. The framework of this paper provides a solution. If you live a miserable life and are even considering committing suicide, you know that you are either close to or have already reached the utility value below which life is no longer worth living. In other words, you know as a fundamental higher-order fact that the range of relevant utility values is finite and your momentary situation approximates the lowest endurable utility value. As a result, you know that whatever you do, it cannot get much worse because if it does get worse, you will terminate your life. At the same time, since your momentary situation is so dire, changing it could improve your life substantially. Therefore, the lowest relevant utility value of transition is closer to the utility value of not transition than the highest relevant utility value of transition. If you then apply a uniform probability distribution due to the Principle of Indifference, you see that the expected utility of transition is higher than that of not transition. Consequently, trans people that are in such a situation can rationally choose to transition because the potential to worsen the overall situation is much more limited than the potential to improve it.

Does the presence of personal transformation change anything about the rationality and authenticity behind choosing to transition? It does not, because on the one hand, the fundamental higher-order fact on which the decision is based, namely the finite range of relevant utility values, applies to the present self as well as all possible future selves. Thus, your future transitioned-self is not completely psychologically alien to you which enables authentic decision-making (as long as your choice is solely based on this higher-order fact).^23^ On the other hand, if you know that you are close to the lowest endurable utility value (or already there), a change in preferences can hardly worsen your life but has the chance to improve it. Therefore, if trans people feel the existential wish and need to transition, as many do according to McKinnon, neither the epistemic nor the personal transformation involved in transition prevent choosing to transition from being authentic and rational.

Let us continue with the decision of whether to become a parent or not. Barnes (2015) argues that an agent who has never wanted children and wishes to stay childfree can rationally choose not to have children. According to her, the agent can rationally base this decision on her firm belief that whatever the experience is like, she is fairly sure it is something she does not want (p. 775). Whether Barnes’ argument is sound shall not be discussed here. Yet, the approach of this paper provides another argument for why a person in the situation described by Barnes can rationally choose to stay childfree.

Once again, such a person can choose between becoming a parent and not becoming a parent. We assume that she knows how it is not to have children, and thus, not becoming a parent is non-transformative. Moreover, since she has never wanted children and also wishes to stay childfree, we can assume that this outcome comprises a utility value that is equal to or greater than zero. In contrast, becoming a parent is transformative and involves ambiguous valence. Paul (2014) writes about this decision situation:

[I]f you were able to find out that the range of the values was asymmetric, such that the worst outcomes were very bad, while the best outcomes were merely pretty good, and you were also able to discover the respects in which they were comparable, … you would be rationally permitted to choose to remain childless, because the asymmetric shape of the value space is so heavily tilted towards the negative. … Do we have this information about the shape of the value space? … No. (p. 155)

But I claim that we do have this information. Due to the negativity bias as a fundamental higher-order fact, we know that the lower endpoint of relevant utility values is further from zero than the upper endpoint. The general shape of the utility space in regard to becoming a parent is therefore asymmetrical and tilted towards the negative. Next, since we do not know how probable each of the relevant utility values is (except that they have positive probability) we apply the Principle of Indifference, saying that each relevant utility value is equally probable. In turn, if each relevant utility value is equally probable and the utility space is tilted towards the negative, we can infer that the expected utility of becoming a parent is below zero. Because of that, a person who does not want to have children and thereby does not perceive the outcome of staying childfree as bad can rationally choose to stay childfree.

As in the case of gender transition, the possibility of a personal transformation does not affect the rationality and authenticity behind choosing to stay childfree. The fundamental higher-order fact on which the decision is based, namely the negativity bias, applies to the present self as well as all possible future selves. So, regardless of how parenthood would change you as a person, the general shape of the utility space would still be tilted towards the negative (given that you are restricted to fundamental higher-order facts). This aspect of your future parent-self is first-personally accessible to you and thus can be used so as to reach a decision authentically.^24^ Consequently, under the conditions described in the last paragraph, you can both rationally and authentically choose to stay childfree even if parenthood had led to a personal transformation.

Let us finish with a more difficult configuration. You know how it is to be childless and value it negatively. In other words, you wish to have children and you know that the outcome of not having children is below zero utility. However, it is not so negative that it puts you into an existential crisis as it is the case for non-transitioned trans people. Should you take the risk of becoming a parent given its ambiguous valence? This is where the framework of this paper gets stuck. The two fundamental higher-order facts discussed above are not sufficient to estimate whether the expected utility of becoming a parent is below or above the utility of not becoming a parent. For that, you would need other higher-order facts about becoming a parent. Maybe, you know from experience that things you wished for and later fulfilled provided you positive utility.^25^ This higher-order fact should then also apply to the wish to have children. We leave open whether rational decision-making is possible here.

To summarise, rational transformative decision-making exclusively with the two discussed fundamental higher-order facts is possible in case of two different initial situations: (1) The non-transformative option involves a utility value close to or even at the threshold below which life is no longer worth living. Here, choosing the transformative option is rational because it cannot make the situation much worse but inheres the potential to massively improve it. (2) The non-transformative option involves an outcome whose utility value is equal to or greater than zero. Here, not choosing the transformative option is rational because its general utility space is tilted towards the negative, leading to an expected utility value below zero.

## Conclusion

The approach I present in this paper builds on Pettigrew’s (2020) redescription, yet, unlike Pettigrew’s solution, it does not exclusively build on testimony. Instead, on the one hand, it employs (fundamental) high-order facts which enable us to assess the shape of the utility space and, given they are close enough, also the range of relevant utility values. On the other hand, it attaches importance to the non-transformative alternative that might be part of the choice set and which involves the outcome of not choosing the transformative alternative. In this way, I both circumvent the hardly solvable technical difficulty of Pettigrew’s solution (Isaacs, 2020) and fulfil Paul’s (2015b) request for authentic decision-making. It is thereby worth noting that the main ideas of this paper can already be found in the work of Paul (2014), namely the differentiation between sharky and normal outcomes as well as the usage of higher-order facts. Therefore, my approach should be compatible with Paul’s perspective on transformative experiences and demonstrates that rational decision-making in such a context is often possible.

## Data Availability

Not applicable.

## Footnotes

1 Pettigrew also provides a solution for personally transformative experiences. Yet, we will not further discuss it since this paper mainly focuses on epistemic transformative experiences.

2 Since this paper’s approach only considers transformative decisions it is not applicable to non-chosen transformative experiences such as illness, accidents, or personal loss (cf. Carel & Kidd, 2020; Hole & Selman, 2020).

3 Pettigrew (2020) simply calls this a deliberative realist understanding of decision theory since, for him, credence and utility functions can also be real in case of constructivism as long as the preference ordering is more fundamental than both (which would amount to constructive psychological realism in this paper).

4 This is in contrast to constructive realism, where preferences are conceptionally prior to utilities (Collins, 2015).

5 Admittedly, this is a strong assumption. It is made for the sake of simplicity since otherwise we had to assess and compare both subjective and non-subjective values of each option which limited the clarity of the paper. Besides, I believe that the additional consideration of non-subjective values would not change any of the exemplary decisions used in the paper.

6 It might be objected that credences are also crucial in order to calculate the expected utility of non-transformative options. This is, of course, true. Yet, in case of Pettigrew’s framework, they are even more important because they do not only determine the probability that a certain outcome occurs but also, indirectly, the utility this outcome derives.

7 Theoretically, if we only use integral numbers, the range of all existing utility values is from negative infinite utility to positive infinite utility.

8 For example, Dougherty et al. (2015) argue that credences can also be assessed by observing others that undergo a transformative experience or by inference from similar experiences. The latter will be further discussed when we introduce Paul’s (2014) idea of higher-order facts in Sect. 4.

9 Testimony faces another challenge if it refers to experiences that are also personally transformative. For example, people who provide testimony about parenthood do so as parents, meaning they already personally transformed from being non-parents to parents. Yet, an agent thinking about whether to have children is a non-parent. So, why should testimony of these parents, who obviously are unlike the agent, be relevant to her before undergoing the transformation? The problem is that even if we assume that the agent somehow knows that she will transform into a similar parent as those whose testimony she relies on, at the moment of deciding, her future parent-self is psychologically alien to her since it is not accessible in any first-personal sense. This is why a choice that is based on such testimony is inauthentic (Paul, 2015b).

10 For example, regarding life satisfaction, a neutral outcome would lie on the agent’s life satisfaction baseline (cf. Fujita & Diener, 2005). Importantly, this does not have to be equal to the status quo since the status quo can also lie below or above the life satisfaction baseline.

11 Sharadin (2015) also uses the concept of valence in the context of transformative experiences. Yet, he thinks that agents can regularly form reasonable expectations about whether an outcome’s valence is positive or negative, including the decision to become a parent. I would disagree with that and say that the valence of becoming a parent is ambiguous in the vast majority of cases.

12 This might not be true if you experience chronic pain because, in this case, experiencing no pain might be a positive outcome. So, there are exceptions.

13 This would only be possible if we knew the range of relevant utility values of being a parent and enough probabilities of individual values so as to determine whether the expected utility is above or below zero. For example, if we knew that the range of relevant utility values was from − 5 to 5 and that 5 had a probability of 0.6, we would already know that the expected utility of the outcome was above zero, regardless of how probable the other utility values were.

14 For example, Paul (2014) describes another sharky outcome when she writes: ‘You can know that getting your legs amputated without anesthesia would be painful, even without exploring the contours of that particular cognitive model’ (p. 28). It seems plausible that we should know this due to some higher-order fact such as the fact that cutting your body is painful (hair and nails excluded) and that the deeper the cut is, the more it hurts.

15 In fact, the categories ‘eating apricot’ and ‘eating nectarine’ could also be seen as higher-order categories that subsume every single eating experience of an apricot and a nectarine, respectively.

16 For example, hierarchical Bayesian models can be used to formalise how people anticipate new outcomes with the aid of higher-order facts (Paul, 2014; Tenenbaum et al., 2011).

17 I thank the anonymous reviewers for making this point and for urging me to more clearly outline how higher-order facts help us to restrict the range of relevant utility values.

18 Importantly, in so doing, we do not leave the first personal perspective which enables authentic decision-making despite the use of testimony (cf. Paul, 2014, pp. 164–165).

19 Rozin and Royzman (2001) list four reasons for this negativity bias. First, there is negative potency: positive entities are less salient than equivalent negative entities. Second, negative events involve steeper gradients than positive ones. Put differently, a negative event is more easily perceived as more negative than a positive event as more positive. Third, the combination of a positive and a negative stimulus yield a more negative evaluation than the algebraic sum of each individual stimulus would suggest. As a consequence, the whole is more negative than the sum of its parts, which is called negativity dominance. Fourth, there is negative differentiation, which means that negative entities are more diverse, engage a wider response repertoire, and their conceptualisations are more complex.

20 Of course, it is also possible that all options in a choice set involve outcomes with ambiguous valence. Yet, we neglect such cases in this paper.

21 Ann’s higher-order facts about eating durian might also comprise a probability distribution. In that case, Ann would not apply the Principle of Indifference but use the probability distribution imposed by the higher-order facts.

22 In a footnote, McKinnon states that there are cases where transition has led to a worse outcome but that they are extremely rare. The large majority of trans people say that their life improved by transitioning.

23 This means that in case of both ex ante and ex post preferences, the agent would choose the transformative option whose outcome has ambiguous valence if the non-transformative option involved a utility value that is close to or even at the threshold below which life is no longer worth living.

24 This means that in case of both ex ante and ex post preferences, the agent prefers a non-transformative option involving a utility value of equal to or greater than zero to a transformative option whose range of relevant utility values can only be assessed via fundamental higher-order facts.

25 For example, Werner and Milyavskaya (2018) might provide some empirical evidence for this hypothesis, particularly in case of self-concordant goals and wishes.
